# Triangulating stapling vs functional end-to-end stapling for cervical esophagogastric anastomosis after esophagectomy for thoracic esophageal cancer: study protocol for a randomized controlled trial

**DOI:** 10.1186/s13063-019-3201-2

**Published:** 2019-01-28

**Authors:** Toshiaki Tsuji, Toshiyasu Ojima, Mikihito Nakamori, Masaki Nakamura, Masahiro Katsuda, Keiji Hayata, Junya Kitadani, Shimpei Maruoka, Toshio Shimokawa, Hiroki Yamaue

**Affiliations:** 1 Second Department of Surgery, School of Medicine, Wakayama Medical University, 811-1, Kimiidera, Wakayama, 641-8510 Japan; 2 Clinical Study Center, School of Medicine, Wakayama Medical University, Wakayama, Japan

**Keywords:** Esophageal cancer, Esophagogastric anastomosis, Functional end-to-end anastomosis, Functional end-to-end stapling, Triangulating stapling, Phase III clinical trial

## Abstract

**Background:**

Several studies have reported that the triangulating stapling method decreases the incidence of anastomotic stricture after esophagectomy. Our previous randomized controlled trial, however, could not confirm the superiority of the triangulating stapling (TS) method over the circular stapling (CS) method in terms of postoperative anastomotic stricture rate. Recently, the functional end-to-end stapling (FEES) method for cervical anastomosis after esophagectomy was developed, and lower anastomotic stricture rates with FEES have been reported than for our previously experienced anastomotic methods. To investigate the optimal anastomotic method, we now compare the TS method with the FEES method for cervical anastomosis regarding decrease in anastomotic stricture after esophagectomy for thoracic esophageal cancer.

**Methods:**

This is a randomized, single-center clinical trial designed to examine the superiority of the FEES method over the TS method for esophageal cancer patients. The primary endpoint is reduction of anastomotic stricture of cervical esophagogastric anastomosis within 12 months after esophagectomy. Secondary endpoints include overall postoperative morbidity within the first 12 months after esophagectomy, incidence of anastomotic leakage, aspiration pneumonia, or reflux esophagitis, and quality of life assessment as measured by the FACT-E at 12 months after esophagectomy. The incidence rate of anastomotic stricture of the TS method was 20% and this rate of the FEES method was estimated to be 4% in our preliminary study. We calculated sample size with a beta error of 0.20 and an alpha error of 0.05. We have been enrolling 125 patients in this trial to either the TS group or the FEES group since January 2017.

**Discussion:**

This study should help to define the optimal anastomotic method for cervical esophagogastric anastomosis after esophagectomy in patients with esophageal cancer.

The FEES method, if proven to be superior to the TS method, can be implemented routinely for esophageal cancer patients with gastric-conduit reconstruction after esophagectomy.

**Trial registration:**

University Hospital Medical Information Network Clinical Trial Registry (UMIN 000025632). Registered on 13 January 2017.

**Electronic supplementary material:**

The online version of this article (10.1186/s13063-019-3201-2) contains supplementary material, which is available to authorized users.

## Background

After subtotal esophagectomy for esophageal cancer, anastomosis of the cervical esophagus and other organs is needed, which connects the adjacent digestive tract to repair digestive function. Reconstruction using a gastric conduit is the most common procedure after esophagectomy, although various other anastomotic techniques have been investigated [1–4]. In spite of recent advances in esophagectomy and management after surgery for esophageal cancer, the optimal anastomotic method has not yet been established. Moreover, complications related to anastomosis remain a major concern for surgeons. Our previous randomized controlled trial (RCT) compared the circular stapling (CS) and triangulating stapling (TS) methods in cervical esophagogastric anastomosis, but it could not demonstrate the superiority of the TS method over the CS method [4]. Complications of esophagogastric anastomosis remain frequent and the improvement of anastomosis techniques is thought to reduce complications.

**Fig. 1:**
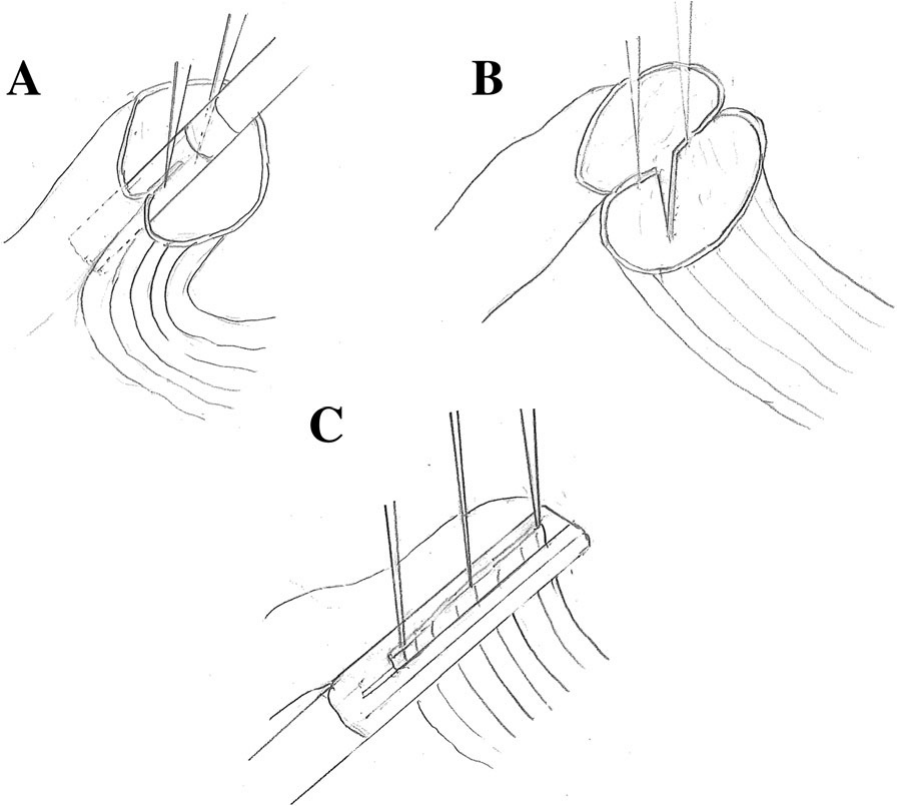
Functional end-to-end stapling for a cervical esophagogastric anastomosis using the linear stapler: **a** The first firing of the stapler was applied to the posterior wall of the remnant esophagus and the gastric conduit after insertion of two suspension sutures in an inverted fashion. **b** Schema after the first firing of the stapler. **c** The second firing of the stapler was applied in an everted fashion

In 1968, Steichen first reported functional end-to-end anastomosis (FEEA) using linear staplers [5]. To date, the FEEA technique has been increasingly adopted in anastomosis of the colon and the intestine. In esophagogastric anastomosis, Collard first reported side-to-side anastomosis using a linear stapler [6], this functional end-to-end stapling (FEES) method and is also known as the “modified Collard anastomosis” [7]. Recently, the FEES method after esophagectomy has been introduced in many facilities. We also have adopted the FEES method for esophagogastric anastomosis (Fig. 1). Theoretically, the FEES method produces a larger anastomosis than other anastomotic methods [6], so we postulate that the incidence of anastomotic stricture for cervical esophagogastric anastomosis using the FEES method should be lower than that using the TS method. No RCTs have yet compared the FEES and TS methods for cervical esophagogastric anastomosis after esophagectomy. To clarify the efficacy of the FEES method, a RCT comparing the FEES method and popular TS method is desirable in esophagogastric anastomosis. Our RCT will compare anastomotic stricture rate and the other postoperative complication rates of the FEES and TS methods for esophagectomy.

## Methods

### Study objectives

This prospective, single-center, phase III trial aims to demonstrate the superiority of the FEES method over the TS method for cervical esophagogastric anastomosis concerning reduction of anastomotic stricture.

### Study setting

This is a **s**ingle-institution, randomized, phase III study.

### Study endpoints

The primary endpoint is the incidence of anastomotic stricture within 12 months after esophagectomy. Secondary endpoints include any incidence of anastomotic leakage, aspiration pneumonia, or reflux esophagitis, overall postoperative morbidity within 12 months after surgery, and quality of life (QOL) evaluation according to the Functional Assessment of Cancer Therapy-Esophageal (FACT-E) version 4.0 12 months after surgery [8]. The Comprehensive Complication Index (CCI) is also calculated as the sum of all complications [9].

### Sample size calculation

Our previous study described a stricture rate of 18% in patients who underwent the TS method [4]. However, as a result of continuing the research involving an additional number of cases, the incidence rate of anastomotic stricture of the TS method was adjusted to 20%.

**Fig. 2:**
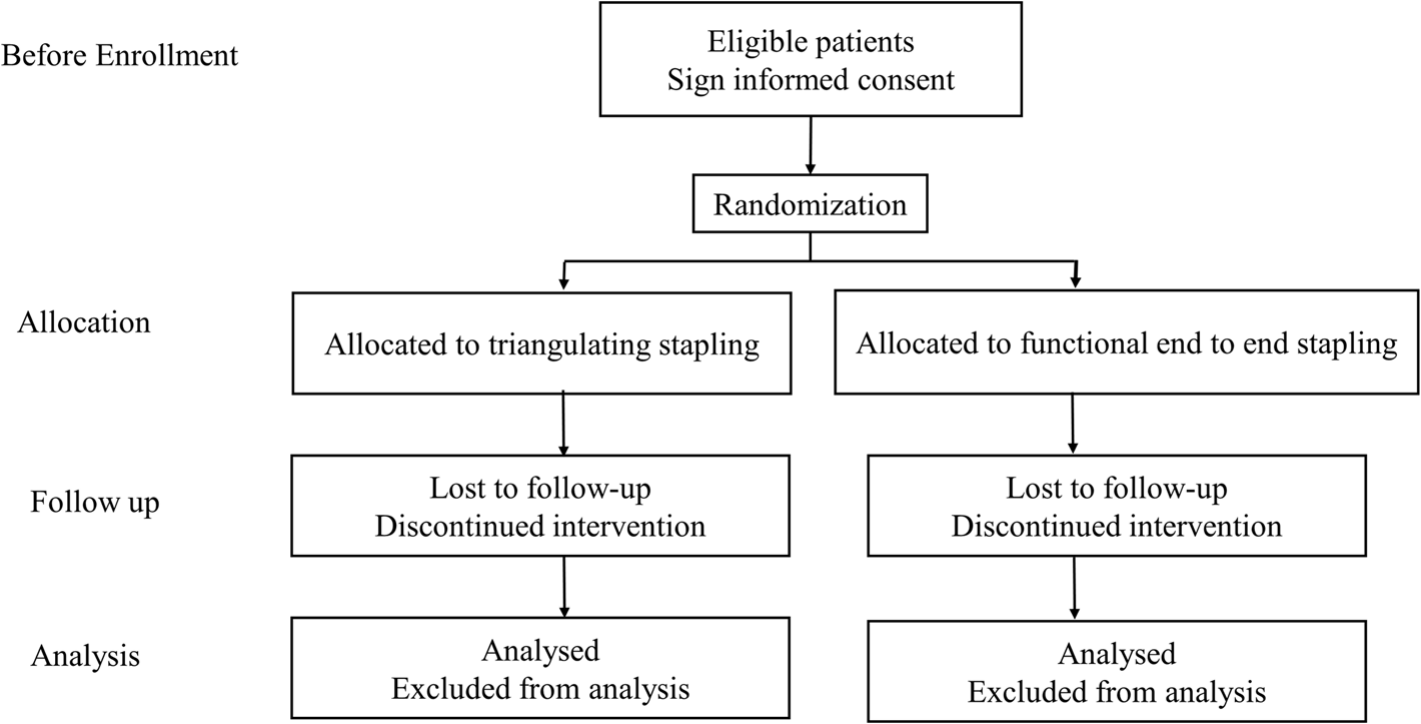
Inclusion and treatment flow diagram

We therefore estimate an incidence rate of 20% in the TS group. This rate of the FEES method was estimated to be 4% in the retrospective study. We hypothesize a reduction in stricture risk from 20 to 4%, and this reduction rate to be a clinically profitable figure. A sample size of 59 patients per arm is needed in this study with a beta error of 0.20 (power 80%) and a one-sided alpha error of 0.05%. Anticipating follow-up loss, we will recruit a total of 125 patients for this trial (Fig. 2).

### Eligibility criteria

#### Inclusion criteria

1. Aged 20 to 80 years
2. Expected curative resection for intrathoracic esophageal cancer
3. Radical esophagectomy with reconstruction using a gastric conduit pulled upward through the posterior mediastinum or retrosternal route
4. Esophagogastric anastomosis at the cervical site
5. Proven written informed consent

#### Exclusion criteria

1. The patients who cannot receive curative resection according to intraoperative findings
2. Inability to undergo either the FEES or TS anastomosis safely according to intraoperative findings (just before cervical anastomosis)
3. Interstitial pneumonia
4. Uncontrollable diabetes mellitus that needs continuous intravenously administered insulin
5. History of myocardial infarction or unstable angina pectoris within 6 months
6. Cardiac failure, New York Heart Association (NYHA) III degree
7. Liver cirrhosis, Child-Pugh class C
8. Active hepatitis
9. Chronic renal failure requiring hemodialysis

### Randomization

After confirmation of the eligibility criteria, intraoperatively, registration is made by telephone to the Central Registry in Wakayama Medical University Hospital (WMUH). Patient randomization in each group is carried out using a series of consecutive numbers and assigned by the WMUH Central Registry. Patients are randomly assigned to either the FEES method group or the TS method group in a 1:1 ratio. Randomization has two stratification factors. The first one is the route of reconstruction (retrosternal or posterior mediastinal route), and the second one is the administration or absence of neoadjuvant chemotherapy. A clinical researcher performs the randomization using computer-generated, random blocks of 4 in the Central Registry of WMUH. Randomization will take place just before cervical anastomosis after feasibility confirmation of both the TS and the FEES method intraoperatively.

### Data collection and statistics

Data will be collected prospectively for all patients including history, physical examination, laboratory data, pathological examination, perioperative clinical information and complications. Data is collected via datasheets on paper and kept securely. All handling cases are managed by subjected identification code or anonymized registration number. In this trial, no loss-to-follow-up patient may be observed within 12 months after surgery, but some patients may develop cancer recurrence. We include patients who die within 12 months of surgery in the analysis. The correspondence table of the anonymizing code and names and the consent form containing the names are kept strictly in separate, lockable, document storage at WMUH. All required parameters will be collected in an SPSS data file (SPSS version 25, IBM statistics, Chicago, IL, USA). All analysis will be done by intention-to-treat in this trial. Statistical analysis of the differences between the TS and FEES groups will be carried out with a chi-square test, Fisher’s exact test, and a Mann-Whitney *U* test. The results of a treatment effect will be estimated with its 95% confidence interval.

### Ethics

The study is conducted in accordance with the Declaration of Helsinki. WMUH Institutional Review Board approved the final version of the protocol (version 1.0) prior to the start of the study (approval number: 1943). This study was registered on the University Hospital Medical Information Network Clinical Trials Registry. (UMIN000025632) (Additional file 1).

### Monitoring

In-house monitoring will be performed every year by a third party to monitor the progress and review the safety and quality of the study.

### Surgical procedure

Patients with thoracic esophageal cancer undergo esophagectomy and mediastinal dissection with extensive lymphadenectomy. Patients undergo thoracoscopic esophagectomy in the prone position. Patients with severe pleural adhesions undergo a right thoracotomy via the fourth intercostal space. After the thoracic procedures are completed, the position of the patient is changed to supine and abdominal lymphadenectomy and gastric conduit reconstruction are performed using either an open laparotomy or laparoscopically. Cervical lymphadenectomy is performed in case of three-field lymphadenectomy. A 4-cm-wide gastric conduit is made along the greater curvature of the stomach using the linear-stapling device Echelon 60–3.5 (Ethicon Endo-Surgery, Cincinnati, OH, USA) in both groups. The gastric conduit is pulled upward to the cervical level through the retrosternal or posterior mediastinal route. At the left side of the neck, esophagogastric anastomosis is performed by either the TS or the FEES method. All operations, including anastomosis, are directed by one of two senior esophageal surgeons (Nakamori or Nakamura). They have experienced 40 cases or more of the TS method and 20 cases or more of the FEES method at the start of this RCT. They will perform both techniques equally.

The TS method is an end-to-end anastomosis using the 60-mm linear-stapling device Echelon 60–3.5 (Endo-Surgery, Cincinnati, OH, USA). The TS method procedure is composed of three steps: the first step is the firing of the linear stapler at the posterior wall of the cervical esophagus and the gastric conduit after inserting three or four suspension sutures. The second step is the firing of the linear stapler at half of the anterior wall after inserting the suspension sutures. The third step is the firing of the linear stapler at the remnant half of the anterior wall as in the second step. This technique has been described previously [4].

The FEES method is a side-to-side anastomosis using the linear-stapling devices Echelon 45–3.5 and Echelon 60–3.5 (Ethicon Endo-Surgery Cincinnati, OH, USA). The posterior wall of the esophageal stump and gastric conduit are placed side by side. Two suspension sutures are placed to secure anastomosis through all layers of the posterior wall of the remnant esophagus and the gastric conduit, then the two forks of the linear stapler Echelon 45–3.5 (Endo-Surgery, Cincinnati, OH, USA) are placed and then fired. After three or four suspension sutures have been placed in the anterior wall of the esophagus and the gastric conduit, the second firing of the stapler is performed for the anterior wall using the Echelon 60–3.5 (Endo-Surgery, Cincinnati, OH, USA) (Fig. 1).

Patients undergo cervical computed tomography (CT) after swallowing water-soluble contrast medium 8 days after surgery. Normal oral intake is allowed after integrity of the anastomosis is confirmed using diagnostic modalities. After discharge, patients are examined at 3, 6, and 12 months after surgery.

### Definition of postoperative complications

Patients routinely undergo postoperative gastrointestinal endoscopy at 12 months or if complaints of symptoms such as dysphagia arise. In this study, anastomotic stricture is a condition that requires balloon dilation of the stenotic anastomosis, with endoscopic proof of a stenosis through which a 9-mm endoscope cannot be passed by esophagogastric anastomosis. Anastomotic leakage is defined as the presence of extraluminal contrast by postoperative CT after swallowing contrast medium, endoscopic visualization of dehiscence or fistula, or flow of saliva or pus through the cervical wound. If pus from the cervical wound with uncertain anastomotic leakage is found, patients undergo a contrast-medium swallow study and a CT study after open drainage of the cervical wound to confirm the existence of anastomotic leakage. Reflux esophagitis is defined as greater than grade A according to the Los Angeles Classification System. Other overall postoperative morbidities are redefined as greater than grade II by the Clavien-Dindo classification [10].

### Follow-up

**Fig. 3:**
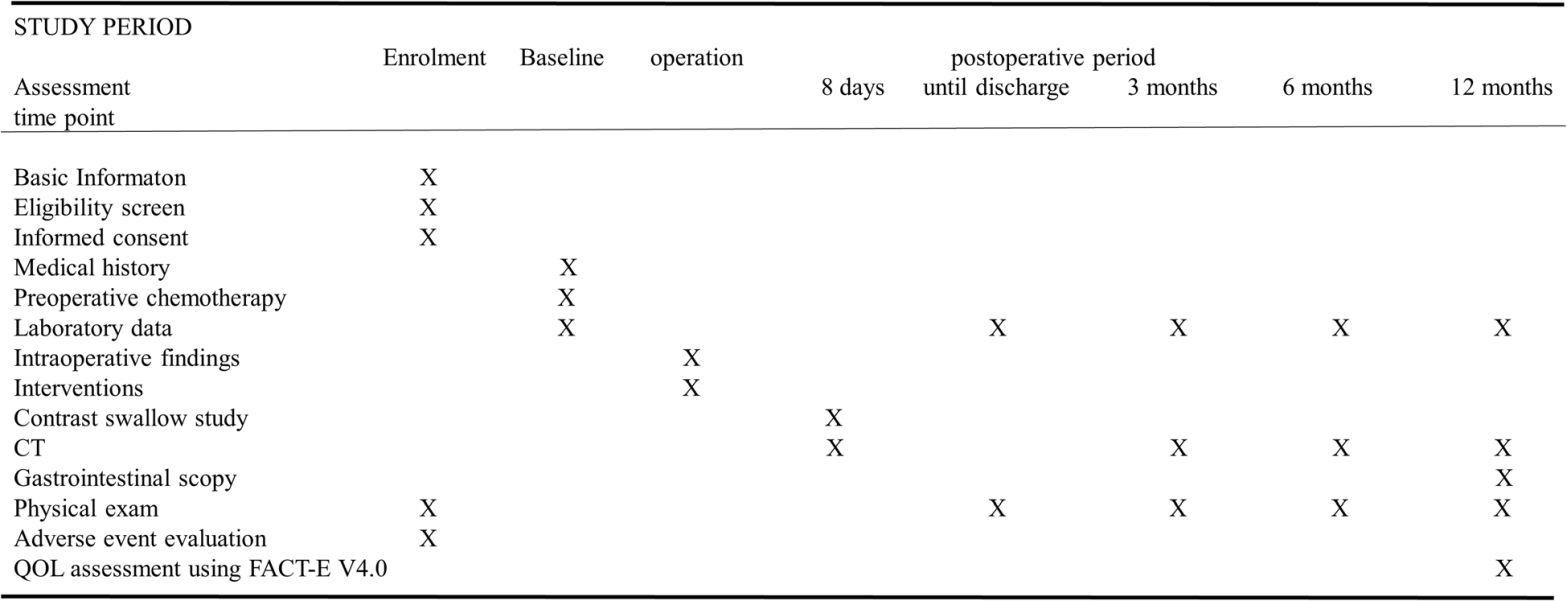
Timeline

Follow-up appointments for all patients will take place at 3, 6, and 12 months following surgery at WMUH. All patients are followed up for 5 years or until death. The enrollment of this trial will be complete by December 2019 and the 12-month follow-up will continue until December 2020 (Fig. 3). The 5-year follow-up of the final participant will be complete by December 2024.

## Discussion

Our previous study comparing the TS method and the CS method showed no significant difference in anastomotic stricture (TS group 18%; CS group 17%; *P* = 0.0935), anastomotic leakage (TS group 2%; CS group 11%; *P* = 0.073), aspiration pneumonia (TS group 6%; CS group 13%; *P* = 0.239), reflux esophagitis (TS group 12%; CS group 13%; *P* = 0.878), or overall morbidity (TS group 69%; CS group 70%; *P* = 0.999) [4].

To investigate the optimal anastomotic method in cervical esophagogastric anastomosis after esophagectomy, we proceed with a further prospective RCT. The reported incidence of anastomotic stricture for the FEES method was lower than for the TS method in cervical esophagogastric anastomosis. This is the first RCT to provide evidence of whether the FEES method reduces the frequency of anastomotic stricture in cervical esophagogastric anastomosis after esophagectomy for esophageal cancer.

The FEES method produces a larger anastomotic area, which is thought to reduce anastomotic stricture. The FEES method is popular in colonic surgery today, but in esophageal surgery the FEES method has not yet become popular and only a few surgeons use this method in esophageal surgery. The FEES method needs only two lots of stapling for esophagogastric anastomosis and can reduce operation time.

## Trial status

The WMUH Institutional Review Board approved the final version of the protocol prior to the start of the study (approval number: 1943). The first patient was recruited on 30 January 2017. Currently, 34 patients have been enrolled in this trial and patient recruitment continues. The projected completion date for this trial is December 2019.

## Additional file

Additional file 1: Standard Protocol Items: Recommendations for Interventional Trials (SPIRIT) 2013 Checklist: recommended items to address in a clinical trial protocol and related documents*. (DOC 124 kb)

## Abbreviations

CS: Circular stapling
CT: Computed tomography
FEEA: Functional end-to-end anastomosis
FEES: Functional end-to-end stapling
RCT: Randomized controlled trial
TS: Triangulating stapling
WMUH: Wakayama Medical University Hospital
